# Supplementary material for: ATP burst is the dominant driver of antibiotic lethality in Mycobacterium smegmatis
Source: eLife. 2026 Jan 13;13:RP99656. doi: 10.7554/eLife.99656 (PMC12799214; doi:10.7554/eLife.99656)
Supplement: MDAR checklist [file elife-99656-mdarchecklist1.docx]

**Materials Design Analysis Reporting (MDAR)**

**Checklist for Authors**

The [MDAR framework](https://osf.io/xfpn4/) establishes a minimum set of requirements in transparent reporting mainly applicable to studies in the life sciences.

*eLife* asks authors to **provide detailed information within their article** to facilitate the interpretation and replication of their work. Authors can also upload supporting materials to comply with relevant reporting guidelines for health-related research (see [EQUATOR Network](http://www.equator-network.org/%20)), life science research (see the [BioSharing Information Resource](http://biosharing.org/)), or animal research (see the [ARRIVE Guidelines](http://www.plosbiology.org/article/info:doi/10.1371/journal.pbio.1000412) and the [STRANGE Framework](https://doi.org/10.1038/d41586-020-01751-5); for details, see *eLife*’s [Journal Policies](https://reviewer.elifesciences.org/author-guide/journal-policies)). Where applicable, authors should refer to any relevant reporting standards materials in this form.

For all that apply, please note **where in the article** the information is provided. Please note that we also collect information about data availability and ethics in the submission form.

**Materials:**

| **Newly created materials** | **Indicate where provided: section/figure legend** | **N/A** |
| --- | --- | --- |
| The manuscript includes a dedicated "materials availability statement" providing transparent disclosure about availability of newly created materials including details on how materials can be accessed and describing any restrictions on access. |  | Not applicable |
|  |  |  |
| **Antibodies** | **Indicate where provided: section/figure legend** | **N/A** |
| For commercial reagents, provide supplier name, catalogue number and [RRID](https://scicrunch.org/resources), if available. |  | Not applicable |
|  |  |  |
| **DNA and RNA sequences** | **Indicate where provided: section/figure legend** | **N/A** |
| Short novel DNA or RNA including primers, probes: Sequences should be included or deposited in a public repository. |  | Not applicable |
|  |  |  |
| **Cell materials** | **Indicate where provided: section/figure legend** | **N/A** |
| Cell lines: Provide species information, strain. Provide accession number in repository OR supplier name, catalog number, clone number, OR RRID. |  | Not applicable |
| Primary cultures: Provide species, strain, sex of origin, genetic modification status. |  | Not applicable |
|  |  |  |
| **Experimental animals** | **Indicate where provided: section/figure legend** | **N/A** |
| Laboratory animals or Model organisms: Provide species, strain, sex, age, genetic modification status. Provide accession number in repository OR supplier name, catalog number, clone number, OR RRID. |  | Not applicable |
| Animal observed in or captured from the field: Provide species, sex, and age where possible. |  | Not applicable |
|  |  |  |
| **Plants and microbes** | **Indicate where provided: section/figure legend** | **N/A** |
| Plants: provide species and strain, ecotype and cultivar where relevant, unique accession number if available, and source (including location for collected wild specimens). |  | Not applicable |
| Microbes: provide species and strain, unique accession number if available, and source. |  | Not applicable |
|  |  |  |
| **Human research participants** | **Indicate where provided: section/figure legend) or state if these demographics were not collected** | **N/A** |
| If collected and within the bounds of privacy constraints report on age, sex, gender and ethnicity for all study participants. |  | Not applicable |

**Design:**

| **Study protocol** | **Indicate where provided: section/figure legend** | **N/A** |
| --- | --- | --- |
| If the study protocol has been pre-registered, provide DOI. For clinical trials, provide the trial registration number OR cite DOI. |  | Not applicable |
|  |  |  |
| **Laboratory protocol** | **Indicate where provided: section/figure legend** | **N/A** |
| Provide DOI OR other citation details if detailed step-by-step protocols are available. |  | Not applicable |
|  |  |  |
| **Experimental study design (statistics details) *** | | |
| **For in vivo studies: State whether and how the following have been done** | **Indicate where provided: section/figure legend. If it could have been done, but was not, write “not done”** | **N/A** |
| Sample size determination |  | Not applicable |
| Randomisation |  | Not applicable |
| Blinding |  | Not applicable |
| Inclusion/exclusion criteria |  | Not applicable |
|  |  |  |
| **Sample definition and in-laboratory replication** | **Indicate where provided: section/figure legend** | **N/A** |
| State number of times the experiment was replicated in the laboratory. | 3 replicates were used |  |
| Define whether data describe technical or biological replicates. | Yes for all experiments |  |
|  |  |  |
| **Ethics** | **Indicate where provided: section/submission form** | **N/A** |
| Studies involving human participants: State details of authority granting ethics approval (IRB or equivalent committee(s), provide reference number for approval. |  | Not applicable |
| Studies involving experimental animals: State details of authority granting ethics approval (IRB or equivalent committee(s), provide reference number for approval. |  | Not applicable |
| Studies involving specimen and field samples: State if relevant permits obtained, provide details of authority approving study; if none were required, explain why. |  | Not applicable |
|  |  |  |
| **Dual Use Research of Concern (DURC)** | **Indicate where provided: section/submission form** | **N/A** |
| If study is subject to dual use research of concern regulations, state the authority granting approval and reference number for the regulatory approval. |  | Not applicable |

**Analysis:**

| **Attrition** | | **Indicate where provided: section/figure legend** | | **N/A** |
| --- | --- | --- | --- | --- |
| Describe whether exclusion criteria were pre-established. Report if sample or data points were omitted from analysis. If yes, report if this was due to attrition or intentional exclusion and provide justification. | |  | | Not applicable |
|  | |  | |  |
| **Statistics** | **Indicate where provided: section/figure legend** | | | **N/A** |
| Describe statistical tests used and justify choice of tests. | **Student's t-test (unpaired, two tailed)** was performed in a pair-wise manner (between control and the desired experiment) as described in the legends for the following Figures-  Figure 2: Differential expression analysis of total proteome upon norfloxacin and streptomycin treatment.  Figure 4: Norfloxacin and streptomycin generate reactive oxygen species as part of their lethality.  Figure 5:Norfloxacin and Streptomycin treatment induces a lethal burst in ATP levels.  Figure 6:Inhibition of excess ATP production mitigates antibiotic lethality.  Figure 7: Norfloxacin and streptomycin induced ROS is insufficient to mediate cell death.  Figure 8:Antibiotic induced ATP levels chelates divalent metal ions.  Figure 9:^13^C metabolomics suggests increased metabolic flexibility and bifurcation of TCA cycle flux as bacterial adaptive mechanism.  Figure 10: Sub-lethal antibiotic exposure can potentiate development of antibiotic resistance.  Figure 4-figure supplement 1  Figure 6-figure supplement 2  Figure 7-figure supplement 1  Figure 9-figure supplement 1  **Benjamini-Hochberg multiple correction test** was applied for results presented in Figures- Figure 3-figure supplement 1 and in the Section- Norfloxacin and streptomycin generate reactive oxygen species to confer cidality | | |  |
|  | |  | |  |
| **Data availability** | | **Indicate where provided: section/submission form** | | **N/A** |
| For newly created and reused datasets, the manuscript includes a data availability statement that provides details for access (or notes restrictions on access). | | Figure 2-figure supplement 1-source data 1A and 1B | |  |
| When newly created datasets are publicly available, provide accession number in repository OR DOI and licensing details where available. | | [Tejan Lodhiya](https://elifesciences.org/reviewed-preprints/99656#x-1083699425), [Aseem Palande](https://elifesciences.org/reviewed-preprints/99656#x-1401083461), Anjali Veeram, [Gerald Larrouy-Maumus](https://elifesciences.org/reviewed-preprints/99656#x85597872), [Dany JV Beste](https://elifesciences.org/reviewed-preprints/99656#x324894013), [Raju Mukherjee](https://elifesciences.org/reviewed-preprints/99656#x1553187311) \|2024\| Label free proteomics in *M.smegmatis* upon exposure to sub-inhibitory concentration of antibiotics  Norfloxacin_7.5hrs \| [doi:10.25345/C5TM72D7D](https://doi.org/doi:10.25345/C5TM72D7D) \|MassIVE MSV000099259  Norfloxacin_15hours\| [doi:10.25345/C59G5GS61](https://doi.org/doi:10.25345/C59G5GS61)\|MassIVE MSV000098909  Norfloxacin_25hrs\| [doi:10.25345/C5QJ78B2Z](https://doi.org/doi:10.25345/C5QJ78B2Z) \|MassIVE MSV000098929  Streptomycin_7.5hrs\| [doi:10.25345/C5JD4Q254](https://doi.org/doi:10.25345/C5JD4Q254)\|MassIVE MSV000098432  Streptomycin_15hrs\|[doi:10.25345/C5T727V26](https://doi.org/doi:10.25345/C5T727V26)\|MassIVE MSV000098761  Streptomycin_25hrs\| [doi:10.25345/C5HQ3SB5Z](https://doi.org/doi:10.25345/C5HQ3SB5Z) \|MassIVE MSV000098771 | |  |
| If reused data is publicly available provide accession number in repository OR DOI, OR URL, OR citation. | |  | | Not applicable |
|  | |  | |  |
| **Code availability** | | | **Indicate where provided: section/figure legend** | **N/A** |
| For any computer code/software/mathematical algorithms essential for replicating the main findings of the study, whether newly generated or re-used, the manuscript includes a data availability statement that provides details for access or notes restrictions. | | |  | Not applicable |
| Where newly generated code is publicly available, provide accession number in repository, OR DOI OR URL and licensing details where available. State any restrictions on code availability or accessibility. | | |  | Not applicable |
| If reused code is publicly available provide accession number in repository OR DOI OR URL, OR citation. | | |  | Not applicable |

**Reporting:**

The MDAR framework recommends adoption of discipline-specific guidelines, established and endorsed through community initiatives.

| **Adherence to community standards** | **Indicate where provided: section/figure legend** | **N/A** |
| --- | --- | --- |
| State if relevant guidelines (e.g., ICMJE, MIBBI, ARRIVE, STRANGE) have been followed, and whether a checklist (e.g., CONSORT, PRISMA, ARRIVE) is provided with the manuscript. |  | Not applicable |

* We provide the following guidance regarding transparent reporting and statistics; we also refer authors to [Ten common statistical mistakes to watch out for when writing or reviewing a manuscript](https://doi.org/10.7554/eLife.48175).

**Sample-size estimation**

- You should state whether an appropriate sample size was computed when the study was being designed
- You should state the statistical method of sample size computation and any required assumptions
- If no explicit power analysis was used, you should describe how you decided what sample (replicate) size (number) to use

**Replicates**

- You should report how often each experiment was performed
- You should include a definition of biological versus technical replication
- The data obtained should be provided and sufficient information should be provided to indicate the number of independent biological and/or technical replicates
- If you encountered any outliers, you should describe how these were handled
- Criteria for exclusion/inclusion of data should be clearly stated
- High-throughput sequence data should be uploaded before submission, with a private link for reviewers provided (these are available from both GEO and ArrayExpress)

**Statistical reporting**

- Statistical analysis methods should be described and justified
- Raw data should be presented in figures whenever informative to do so (typically when N per group is less than 10)
- For each experiment, you should identify the statistical tests used, exact values of N, definitions of center, methods of multiple test correction, and dispersion and precision measures (e.g., mean, median, SD, SEM, confidence intervals; and, for the major substantive results, a measure of effect size (e.g., Pearson's r, Cohen's d)
- Report exact p-values wherever possible alongside the summary statistics and 95% confidence intervals. These should be reported for all key questions and not only when the p-value is less than 0.05.

**Group allocation**

- Indicate how samples were allocated into experimental groups (in the case of clinical studies, please specify allocation to treatment method); if randomization was used, please also state if restricted randomization was applied
- Indicate if masking was used during group allocation, data collection and/or data analysis
